# Looking to the Future: Biomarkers in the Management of Pancreatic Adenocarcinoma

**DOI:** 10.3390/ijms12095895

**Published:** 2011-09-14

**Authors:** Jennifer L. Spratlin, Karen E. Mulder

**Affiliations:** Department of Oncology, Cross Cancer Institute, School of Cancer, Imaging and Engineering Sciences, Faculty of Medicine and Dentistry, University of Alberta, 11560 University Avenue, Edmonton, Alberta, T6G 1Z2, Canada

**Keywords:** pancreatic adenocarcinoma, hENT1, hCNT3, SPARC, cDK, biomarker, clinical trials, gemcitabine, oxaliplatin

## Abstract

The incidence and mortality of pancreas cancer converge. There has been little advancement in the treatment of pancreas cancer since the acceptance of gemcitabine as the standard therapy. Unfortunately, the efficacy of gemcitabine is dismal. While there is much discussion for the development of biomarkers to help direct therapy in this area, there is little action to move them into clinical practice. Herein, we review potential pancreatic cancer biomarkers and discuss the limitations in their implementation.

## 1. Introduction

Adenocarcinoma of the pancreas is one of the most devastating cancers. More than 44,000 patients in the United States will be diagnosed with pancreas cancer in 2011 and almost 38,000 will die in the same year [1]. With the yearly incidence rate almost equaling the yearly mortality rate, the diagnosis of pancreas cancer is almost always fatal. Adenocarcinoma of the pancreas is clinically divided into three subgroups at diagnosis: resectable (~10–20%), locally advanced unresectable (~30–40%), and metastatic (~50%). The only chance of a cure is with complete surgical resection, however the understood cure rate in that patient population is still only 5%. On average, patients with resectable, locally advanced unresectable, and metastatic pancreas cancer survive 23, 6–12, and ~6 months, respectively [2,3]. Despite hundreds of pre-clinical and clinical trials, there are still only limited treatment options for pancreatic cancer patients. The adjuvant treatment of pancreatic adenocarcinoma is limited to the use of either 5-fluorouracil (5FU) or gemcitabine. These have been proven to have equal efficacy once tumors are surgically resected [4]. In the metastatic setting, there is an ongoing struggle to identify new and better treatment options for patients. Clearly, with a historical average overall survival of approximately 6 months, therapeutic options require improvement.

Since the pivotal first-line clinical trial published in 1997, gemcitabine (2′,2′-difluorodeoxycytidine) has been considered the mainstay therapy for advanced disease [3]. This trial randomized 126 patients to gemcitabine or 5FU and resulted in an improvement in clinical benefit, a composite endpoint consisting of improvement in pain, performance status, and weight maintenance. Though the gain in median survival was statistically significant, the benefit was only 1.24 months. Hundreds of clinical trials investigating both standard cytotoxics and targeted agents have since been undertaken, almost all of which have failed. Proof of concept for the utility of biologics in advanced pancreas cancer occurred in 2007 when erlotinib, a small molecule inhibitor of the epidermal growth factor receptor (EGFR) pathway, in combination with gemcitabine improved survival for patients over gemcitabine alone [5]. The OS advantage with the addition of erlotinib, however, was less than 2 weeks and, as such, it is not widely considered clinically relevant. Other targeted agents, including but not limited to, small molecule and antibody directed inhibition of angiogenesis, antibody mediated inhibition of EGFR, and inhibiting mammalian target of rapamycin (mTOR) have been unsuccessful in phase II and phase III clinical trials [6–14]. Furthermore, the combination of more than one biologic has not been successful [15].

More recently, oxaliplatin-based cytotoxic therapy has emerged as a potential treatment option both in the first and second line setting for metastatic pancreas cancer. First line, the combination of 5FU, leucovorin, irinotecan, and oxaliplatin, known commonly as FOLFIRINOX, improves progression free survival and OS compared to single agent gemcitabine [16]. These results come with the caveat of significantly increased toxicity including grade 3 or 4 neutropenia, febrile neutropenia, thrombocytopenia, diarrhea, and sensory neuropathy. The study did not include patients older than 75. The publication clearly states FOLFIRINOX should only be administered to patients with good performance status [16]. Second line oxaliplatin based treatment in combination with 5FU and leucovorin as part of a regimen, abbreviated as OFF, has also shown at least a 2 month OS benefit after gemcitabine failure [17]. Again, this trial was limited to a highly selected patient population with continued good performance status despite progressing on first line therapy.

Due to the limited benefit of gemcitabine in an unselected patient population and the high level of potential toxicity with oxaliplatin based chemotherapy regimens, there is a desperate need to identify potential biomarkers in the treatment of pancreas adenocarcinoma. This review will focus on the potential current relevant biomarkers which may be predictive or prognostic of cytotoxic chemotherapies currently being used in metastatic pancreas cancer including biomarkers.

## 2. Gemcitabine Factors

Although FOLFIRINOX offers improved PFS and OS over single agent gemcitabine, it is not possible to offer this treatment to all patients due to its toxicities. As such, gemcitabine remains the clinical reference agent for the treatment of metastatic pancreas cancer as well as one of the only two options for treatment in the adjuvant setting. To date, despite leading experts stating their opinion that personalized medicine and chemotherapeutic options should take top priority in clinical trials, there have been no trials dedicated to the validation of a predictive or prognostic biomarker for gemcitabine utility. Gemcitabine is a pyrimidine nucleoside analog with activity against a number of tumor types [18]. Gemcitabine exerts its cytotoxic effects inside the cell where kinases phosphorylate it to mono-, di-, and tri-phospate forms, the latter of the two being the active metabolites. Gemcitabine is self-potentiating with multiple mechanisms of action including DNA chain termination, induction of apoptosis, and DNA synthesis inhibition [19–21]. Once incorporated by deoxyribonucleic acid (DNA) and ribonucleic acid (RNA) gemcitabine is protected from base excision repair [19,22].

As a hydrophilic compound, gemcitabine diffusion across the plasma membrane is difficult without the use of physiologic nucleoside transporter proteins. There are two known categories of nucleoside transporters: equilibrative (ENT) and concentrative (CNT) [23]. Both have data supporting their use as a biomarker to direct gemcitabine treatment.

### 2.1. hENT1

There are two human equilibrative nucleoside transporters (hENT), hENT1 and hENT2, which are found in most cell types and mediate concentration dependent gemcitabine cellular uptake [24]. These transmembrane proteins are differentiated functionally by their sensitivity to nitrobenzylmercaptopurine ribonucleoside (NBMPR) with hENT1 being sensitive while hENT2 is not [25]. There is mounting evidence that hENT1 is a predictive and prognostic biomarker for the use of gemcitabine both in the adjuvant and metastatic setting. In 2004, hENT1 abundance in biopsies of patients that had been treated with gemcitabine for their advanced disease was retrospectively determined by immunohistochemistry [26]. The difference in overall survival between those patients that had uniform hENT1 on their tumors compared to those that did not have uniform hENT1 staining was 13 versus 4 months (*p* = 0.01). This initial study prompted further investigation into hENT1 as a biomarker to guide gemcitabine use.

*In vitro*, higher levels of hENT1 measured by quantitative RT-PCR increased gemcitabine sensitivity [27]. Three common pancreas cancer cell lines had variable hENT1 expression and those with the most hENT1 were the most sensitive to gemcitabine cytotoxicity. Similar results have been seen in several *in vivo* retrospective assessments of the hENT1 in the setting of gemcitabine treatment. In both univariate and multivariate models, hENT1 expression via immunohistochemistry assessment on tissue microarrays was associated with improvements in OS and DFS in patients treated adjuvantly with gemcitabine [28]. In this study, 91 patients were treated on the gemcitabine arm with significant benefit seen in OS and DFS when low versus high, and high versus no hENT1 grouping were assessed. The same findings were not seen in the 5FU treated population. Further, evaluation hENT1 using standard immunohistochemistry methods of 45 patients who received gemcitabine treatment confirms significantly longer DFS and OS with high hENT1 than with low hENT1. The median DFS and OS were 8.4 versus 46.8 months and 13.3 versus not yet reached at the time of publication for low versus high hENT1, respectfully [29]. Finally, 434 pancreas cancer patients have been evaluated for hENT1, 243 of which received gemcitabine chemotherapy [30]. Overall survival was associated with gemcitabine and high hENT1 even when corrected for tumor grade, tumor size, lymph node status, and resection margins. There was no difference in OS between high and low hENT1 in those patients not exposed to gemcitabine.

Transcriptional analysis of hENT1 has also been proven to be a prognostic tool [31]. In 102 evaluated patients, those patients with high hENT1 had significantly longer OS and disease-free survival as well as a longer time to progression. Multivariate analysis demonstrated hENT1 as an independent prognostic factor.

### 2.2. hCNT3

The other gemcitabine membrane transporters are concentrative in nature. Human concentrative nucleoside transporter 1 (hCNT1), 2 (hCNT2), and 3 (hCNT3) use the sodium gradient to move gemcitabine across the plasma membrane against the concentration gradient [24]. Ubiquitous, hCNT3 is present in most tissues and is responsible for the majority of gemcitabine transport by the CNTs. There is not as much information in the medical literature regarding the use of hCNT3 as a biomarker in pancreas cancer. Maréchal *et al.* evaluated hCNT3 alone and in combination with hENT1 [29]. Alone, high versus low hCNT3, as determined by standard immunohistochemistry methods, was associated with improvement in OS (not yet reached at the time of publication versus 21.6 months). Disease recurrence was increased (8.6 versus 23.5 months, *p* = 0.02) and three year survival was less (26.1% versus 54.6%, *p* = 0.028) in the hENT1 low versus high group. When hCNT3 is combined with hENT1, the three year survival jumps to 81.1% suggesting the use of the two biomarkers in combination may be more robust than using one alone.

### 2.3. dCK

Deoxycytidine kinase (dCK) is the enzyme responsible for the rate limiting step which converts administered gemcitabine to its active metabolites. An abundance of dCK, or an increase in its activity will increase the active forms of gemcitabine thereby potentially increasing gemcitabine efficacy [32]. Pre-clinically, a composite of hENT1, dCK, ribonucleoside-diphosphate reductase M1 (RRM1), and RRM2, the latter two also being involved in gemcitabine transport and metabolism, indicates gemcitabine sensitivity or resistance in eight pancreatic cell lines [33]. *In vivo*, high dCK mRNA levels in 70 patients who received palliative gemcitabine treatment correlated with significantly longer DFS [34]. In a larger patient population of 243 patients treated with adjuvant gemcitabine-based treatment after resection of their tumors, a significant interaction was seen with benefit in OS and dCK protein expression [30].

## 3. Tumor & Matrix Factors

### 3.1. SPARC

Secreted protein acidic and rich in cysteine (SPARC), also called osteonectin, is a protein located both on pancreas cancer tumor cells and fibroblasts within the peritumoral extracelllular matrix. It is involved in many tumor related functions including adhesion, remodeling, angiogenesis, and cell migration and invasion [35–38]. SPARC has also been shown to have a role in growth rate modulation as demonstrated by SPARC knockout mice having increased rate of tumor growth than those mice with intact SPARC [39,40]. The abundance of SPARC on pancreas cancer cells and matrix fibroblast varies with tumors often losing SPARC expression due to epigenetic changes; this does not appear to be the case with fibroblasts [41].

SPARC is emerging as a possible prognostic biomarker and has in a small patient population, demonstrated predictive capabilities in terms of being able to direct treatment decisions in pancreas adenocarcinoma. Multiple studies have demonstrated SPARC overexpression in a variety of cancers confers a poor prognosis [42–45]. In esophageal and gastric tumors, high SPARC is associated with lymph node metastases and worse clinical outcome [42,45]. In breast cancer, SPARC was found to be present in higher quantities than normal breast tissue and correlated with lymph node metastases, tumor grade, and 10-year survival [43]. In bladder cancer, there again was a connection between high SPARC and poor prognosis but additionally, gene expression of SPARC correlated with matrix metalloproteinase-2 (MMP-2) expression [44]. MMP-2 is a matrix protein known to have roles in tissue breakdown, remodeling, and metastasis. This interaction may be a clue as to SPARC importance in patient clinical outcomes.

In pancreas cancer, it is becoming clear that the location of SPARC overexpression is important. Infante *et al.* published data demonstrating it is the SPARC in pancreatic cancer stromal fibroblasts that confers a worse prognosis and not the SPARC on pancreatic tumor cells proper [46]. Patients whose tumors stoma was positive versus negative for SPARC had median OS of 15 versus 30months (*p* < 0.001); the same was not seen when comparing tumor cell SPARC. Early data also suggests a potential role to predict response to nab-paclitaxel, a drug currently under investigation in combination with gemcitabine for the treatment of advanced pancreas cancer [47]. This will be important if the ongoing phase III clinical trial portrays a survival advantage of the combination over single agent gemcitabine (NCT00398086).

### 3.2. Prostate Stem Cell Antigen

Prostate stem cell antigen (PSCA) is a cell surface protein present in about 70% of pancreas cancers with normal tissue expression being low [48,49]. PSCA copy number in the blood of patients with pancreas cancer compared to normal patients assessed with real-time quantitative polymerase chain reaction was significantly elevated [50]. Pre-clinical xenografts mouse models had inhibition of tumor growth and decrease in distant metastases when exposed to an anti-PSCA mouse monoclonal antibody [51]. Anti-PSCA treatment also resulted in a decrease of tumor growth initiation in a pancreatic cancer xenograft model using the Capan-1 cell line [52]. AGS-1C4D4 is a fully human IgG_1κ_ monoclonal antibody against PSCA. A recent clinical trial (NCT00902291) reported an improved 6 month survival rate in patients with PSCA positive tumors in patients receiving the combination of AGS-1C4D4 + gemcitabine versus gemcitabine alone [53]. Together, these data suggest PSCA is a negative prognostic feature for pancreas cancer and a possible predictor of anti-PSCA therapy.

## 4. Other Factors

There are a few other potential pancreatic cancer biomarkers that may have clinical impact in the future though none are as robust as those discussed above. Tissue microarrays have been shown to have potential utility to identify possible patterns associated with pancreas cancer, possibly leading to the discovery of a gene or protein that might be a predictive or prognostic tool [54–57]. Other possible markers in early development include the following: *in vitro*, activated leukocyte cell adhesion molecule (ALCAM, CD166) as a marker of cell adhesion and chemoresistance [58]; pancreatic stellate cells (PSCs), isolated according to CD10 surface expression, are associated with lymph node status, increased invasiveness, and decreased survival [59]; lack of or weak nuclear staining of the basic helix-loop-helix domain containing class-B2 transcriptional factor BHLHB2 which is induced by hypoxia, a common finding in pancreas cancer, confers a median survival of 13 months compared to 27 months with strong staining (*p* = 0.03) and is touted as an independent prognostic factor for survival [60]; and finally, Dkk-3 (a member of the Wnt signaling cascade and a possible tumor suppressor) overexpression correlates with tumor growth inhibition after gemcitabine or 5FU exposure and as such may be a predictor of chemotherapy efficacy [61].

The targeting of angiogenesis has not been a successful endeavor for the treatment of pancreas cancer. Noted above, clinical trials using either monoclonal antibodies or small molecule inhibitors of the vascular endothelial growth factor (VEGF) receptor (VEGFR) pathway have failed to demonstrate benefit [6–10]. To the contrary, pre-clinical data supports the effectiveness of inhibiting the VEGF family with a halting of tumor growth likely mediated by decreasing neovascularization and lymphangiogenesis while increasing meaningful delivery of cytotoxic drugs [62,63]. A possible prognostic test, higher pre-treatment serum VEGF/soluble VEGFR-1 ratio is associated with worse survival in patients with pancreas cancer [64]. In addition, and not discussed in this review, there exists a whole body of literature supporting the use of these agents and possible mechanisms of resistance to them in pancreas cancer. Unfortunately, positive findings linking antiangiogenic agents and pancreas cancer are meaningless if the drugs targeting this pathway are ineffective in the clinical setting.

## 5. Conclusions

In a consensus report from the National Cancer Institute clinical trials planning committee on pancreas cancer treatment, experts put a focus on the discovery, development, and validation of one or more biomarkers for this deadly disease [65]. After all, wouldn’t it be ideal to explain to a patient that, based on a biomarker in their tumor, they should be treated with drug A and not drug B, or vice versa. Similarly, if a metastatic patient with performance status that does not allow for delivery of FOLFIRINOX was in clinic, would it not be important to know that a biomarker can determine whether they will benefit from gemcitabine, as if not, their best option may be to control symptoms in the last few months of their lives without chemotherapy. To date, despite leading experts stating their opinion that personalized medicine and chemotherapeutic options should take priority in clinical trials, there have been no trials dedicated to the validation of a predictive or prognostic biomarker for gemcitabine, 5FU, or oxaliplatin-based chemotherapy utility in pancreas cancer. Perhaps the reasons include the sense that we are still not at the point in pancreas cancer to have a robust enough biomarker data to determine efficacy of one or more of our active drugs or perhaps it is because clinical trials are still driven by financial considerations and profit margins for drug company sponsors. One could challenge that line of thinking as we have not made much in the way of progress to date by simply guessing and testing a new drug or a drug combination.

At this time, hENT1, hCNT3, SPARC and PSCA are the most clinically advanced biomarkers. They have all been investigated retrospectively and are shown to have some utility associated with gemcitabine, nab-paclitaxel, or AGS-1C4D4 treatment. Unfortunately there is little information regarding biomarkers for FOLFIRINOX at the current time. One could argue that, though this is now considered the reference combination for patients, in reality, its generalized clinical use is difficult without modifications to the regimen in one form or another.

The bottom line is that we need better treatments for pancreas cancer and biomarkers should be used both retrospectively and a priori in future clinical trials. One method of achieving this is to select a patient population via one or more biomarkers to guide therapy decisions. Our group has recently received grant funding from the Alberta Health Innovates Health Solutions and the Alberta Cancer Foundation where we will integrate a priori biomarker testing in clinical trials for both the adjuvant and metastatic pancreas cancer patient population. To our knowledge this is a relatively novel course in pancreas cancer clinical trials and one that should be actively pursued. The goal is to personalize the treatment for our pancreas cancer patients for better outcomes and more tolerable toxicities.
